# In-school adolescents’ weight status and blood pressure profile in South-western Nigeria: urban-rural comparison

**DOI:** 10.1186/s40608-018-0179-3

**Published:** 2018-01-26

**Authors:** Akinlolu Gabriel Omisore, Bridget Omisore, Emmanuel Akintunde Abioye-Kuteyi, Ibrahim Sebutu Bello, Samuel Anu Olowookere

**Affiliations:** 1 Department of Community Medicine, Osun State University, Osogbo, Nigeria; 2 Department of Family Medicine, Obafemi Awolowo University Teaching Hospitals Complex, Ile-Ife, Nigeria; 3 Department of Community Health, Obafemi Awolowo University Ile-Ife, Ile-Ife, Nigeria

**Keywords:** Blood pressure, Body mass index (BMI), Obesity, Urban, Rural

## Abstract

**Background:**

Obesity is a risk factor for hypertension. The study observed the relationship between adolescent weight status and blood pressure (BP) and the determinants of the BP pattern in urban and rural areas.

**Methods:**

This was a cross-sectional study of 1000 randomly selected respondents (500 from urban and 500 from rural areas) who had anthropometry and BP measurements done. The pattern of BP measurements based on the weight status by location was observed. Statistical inferences were drawn via Chi-square and logistic regression.

**Results:**

The mean age for all the respondents was 13.73 years ±2.04 (13.63 ± 2.05 for urban and 13.82 ± 2.03 for rural). Systolic and diastolic BP generally increased with increasing respondents’ age, with mean pressures higher in urban areas. About 3% were obese, while 7.7% were overweight. The overall prevalence of high BP was 4.1%, with two-thirds coming from urban areas. On logistic regression analysis, the significant variables associated with high BP include being female (AOR 2.067, 95%CI1.007–4.243, *p* = 0.048), overweight (AOR 5.574, 95%CI 2.501–12.421, *p* = 0.0001) and obese (AOR 12.437, 95%CI 4.636–33.364, p = 0.0001).

**Conclusion:**

High BP was associated with being female, overweight and obesity in both urban and rural areas. Urgent measures are needed to address increasing prevalence of overweight and obesity among adolescents and consequent high blood pressure.

## Background

Obesity had previously been regarded as a problem of the developed world and that of adults, however current statistics indicate that overweight and obesity are increasingly common in developing countries and among children and adolescents [1–3]. Obesity accounts for the rising incidence of hypertension among adolescents worldwide and hypertension among adolescents is frequently predictive of future adult hypertension, with its attendant morbidity and mortality and reduced life expectancy [4]. Lifestyle diseases or chronic Non-Communicable Diseases (NCDs) like hypertension, diabetes mellitus, coronary artery diseases and stroke in adults have been related to the preponderance of risk factors in childhood and adolescence [5].

Of all the NCDs, hypertension is the most prevalent and commonest cause of morbidity and mortality [6]. It is also the commonest risk factor for cardiovascular disease (CVD) [7]. Hypertension occasionally begins in adolescence or childhood and tracks into adulthood [8]. The increasing prevalence of obesity among adolescents and children worldwide has been associated with increased prevalence of hypertension. However, more evidence on the relationship between adolescent weight status and blood pressure (BP) patterns is required despite studies done previously in Nigeria [9–11].

Previously adult obesity and high BP definitions have been used for adolescents but such are no longer recommended. The BMI changes during childhood differ between boys and girls, so age and sex-specific reference data (centile cut off points on charts) such as that of the International Obesity Task Force (IOTF) are necessary to interpret the measurement [12]. Similarly, the Fourth Report on the Diagnosis, Evaluation, and Treatment of High BP in Children and Adolescents recommends age-, sex-, and height-specific values that are at >95th percentile of normative systolic and diastolic BP values as diagnostic of high BP in childhood [13].

Apart from using International reference standards to measure obesity and high BP, an additional contribution to knowledge being made by this study is that it was conducted in both urban and rural areas. Few studies have attempted to look at adolescent weight status and relate it to the BP pattern and predictors among urban and rural adolescents in Nigeria. Since the relationship between increasing weight status and high BP has been established, [7, 10] and that BP during childhood and adolescence is a recognized predictor of adulthood BP, leading to increased CVD mortality, [14] it becomes imperative to study the weight status and BP profile of adolescents who are gradually moving into adulthood not only in semi-urban or urban areas where more studies have been done but also in rural areas [9, 10, 15]. Because of the different characteristics of urban and rural dwellers in terms of family values, dietary pattern, physical activity and exposure to social amenities, the pattern of obesity/hypertensive range blood pressure is expected to be different between the two areas. Thus, it is expected that the prevalence and determining factors of obesity/ hypertensive range blood pressure may differ between rural and urban areas, hence the comparative nature of this study. This is a very important consideration in determining intervention strategies to apply to both rural and urban settings. This study, therefore, aims to examine in-school adolescents’ weight status and relate it to the BP profile in both urban and rural areas of Osun State, South Western Nigeria as well as identify probable predictors for the BP pattern.

## Methods

The study was carried out in Osun State, one of the six states in south-western Nigeria in the year 2012 and primary data was independently collected by the authors. The State has three senatorial districts, each with two health zones and 10 local government areas (LGA). Osun East senatorial district was selected by simple random sampling out of the three senatorial districts. The minimum sample size was determined by using the formula for calculating sample size for the comparison of two independent proportions using urban and rural prevalence of 20.9% and 11.9% respectively from a previous study [16]. The study population included 500 students from urban and 500 from rural areas making a total population of 1000 students from eight schools (four schools from urban areas and four from rural areas) across two health zones in the Osun East senatorial district selected by a multi-stage sampling method. Two communities, one urban and one rural, were chosen by simple random sampling from each of the two health zones in the Osun East senatorial district and two co-education schools were then chosen by simple random sampling from each community. The students in selected schools were stratified by arm and sex and a sample proportionate to the school population and the male-female distribution was studied from each arm. Pre-tested and pre-coded structured questionnaires were completed by the selected 1000 respondents and anthropometry (height and weight) and BP measurements were done.

The weight was measured to the nearest 0.1 kg with a bathroom weighing scale of the United Nation Children Fund (UNICEF) type and the height was measured to the nearest 0.5 cm using a stadiometer. The weight and height were measured with the respondents in light clothing. [16, 17] The BP measurements were taken with Accoson mercury sphygmomanometers with appropriate size cuff. The BP was measured with the adolescent in a sitting position, with the arm at the level of the heart. The cuff was inflated to a level at which the distal arterial pulse was not palpable. It was then deflated at a rate of 2–3 mmHg per second. Systolic blood pressure (SBP) was recorded on hearing the first sound (phase I), while Diastolic blood pressure (DBP) was taken on complete disappearance of Korotkoff sounds phase V [18]. Two measurements were taken with the patient in sitting position by the same (two) trained members of the research team and the average of the two was taken as the final measurement. In cases where there were differences of up to 10 mmHg in either of the systolic or diastolic readings, a third and final measurement was taken by the lead researcher and this was taken as the final measurement. Adolescents were classified as having normal BP, Pre-Hypertension and Hypertension corresponding to less than 85th percentile, 85th -95th percentile and above 95th percentile according to the Fourth Report on the Diagnosis, Evaluation, and Treatment of High BP in Children and Adolescents [13].

From the height and weight measurement, the Body Mass Index (BMI) of each respondent was calculated. The BMI was calculated using the formula- Weight (kg)/Height (m2). The calculated BMI for each of the respondent in the study was compared with the IOTF age and sex-specific values before any of the study respondents were classified as normal weight, overweight or obese. The research team was made up of qualified and trained research assistants consisting of medical doctors, nurses, medical students and laboratory personnel. They were trained adequately on the study instruments and procedures prior to data collection.

The data obtained were entered and analysed using SPSS 16. Relevant descriptive and inferential statistics were done. Chi-square test and binary logistic regression were used for bivariate analysis. Multivariate analysis using binary logistic regression was used to evaluate variables that were independently associated with high BP. Criteria for inclusion of variables in the logistic regression model was a *p*-value of < 0.2 in the bivariate. Odds ratios (OR) and 95% confidence intervals (CI) were presented and used as measures of association. p-value < 0.05 was regarded as statistically significant.

The main outcome measures were the systolic and diastolic BP of participants as well as the BMI using the IOTF age and sex specific cut off points. Physical activity was derived from a standardised instrument recommended for adolescents [19]. The Moderate to Vigorous Physical Activity (MVPA) measure of the instrument was used for this study [19]. The measure assessed the number of days’ subjects had accumulated 60 min of MVPA during the past 7 days and for a typical week. The measure defined physical activity broadly as "one that at least increases your heart rate and makes you get out of breath some of the time". An average of the 2 weeks (last 7 days plus a typical week) yielded a score of days per week the adolescent had at least 60 min of MVPA per day. Five or more days per week met the guideline of being physically active. Socio-economic status (SES) was classified into two (high and low SES) based on a modified wealth index approach [20]. The modified wealth index approach used in this study was based on an eight-item index of household assets including means of mobility such as the possession of motorcycles and cars. House type (size and whether rented or owned) and type of walls/floor materials, as well as toilet facilities available, were taken into consideration. The estimated pocket/feeding money given to the adolescents were also considered. The minimum and maximum obtainable score from the modified index was 0–20, with a mean as well as a median score of 14.00 indicating a normally distributed sample. Respondents who had a score above 14 were regrouped as belonging to high socio-economic class while those who had a score of 14 and below were regrouped as belonging to low socio-economic class (SES) based on the average score (20).

## Results

Table 1 shows the socio-demographic characteristics of respondents. Most of the respondents in both urban and rural schools were in the age group 10–13 (early adolescence) and 14–16 years (middle adolescence). The mean age (SD) for all the respondents was 13.73 (2.04) years; for urban 13.63 (2.05) and for rural 13.82 (2.03) years. Male respondents were 51.0% in both urban and rural schools. Of the 1000 respondents, higher proportions were from more highly educated parents (paternal 65.1%, maternal 60.6%) compared with their respective counterparts. The table also shows that significantly higher proportions of respondents in urban schools were in private schools and from more highly educated parents and high socioeconomic status compared with their rural counterparts.

**Table 1:** Socio-demographic characteristics of respondents

Variable	Urban *N* = 500 (%)	Rural *N* = 500 (%)	Total *N* = 1000 (%)	Remark
Age Group				
10–13 Years	230(46.0%)	225(45.0%)	455(45.5%)	χ^2^ = 1.56, *p* = 0.459
14–16 Years	233(46.6%)	227(45.4%)	460(46.0%)	
17–19 Years	37(7.4%)	48(9.6%)	85(8.5%)	
Sex				
Male	255(51.0%)	255(51.0%)	510(51.0%)	χ^2^ = 0.001, *p* = 1.00
Female	245(49.0%)	245(49.0%)	490(49.0%)	
Ethnicity				
Yoruba	458(91.6%)	477(95.4%)	935(93.5%)	χ^2^ = 14.32, *p* = 0.003
Igbo	38(7.6%)	14(2.8%)	52(5.2%)	
Hausa	1(0.2%)	4(0.8%)	5(0.5%)	
Others	3(0.6%)	5(1.0%)	8(0.8%)	
Father’s Educational Status				
Low^a^	60(12.0%)	115(23.0%)	175(17.5%)	χ^2^ = 20.97, *p* < 0.001
High^b^	348(69.6%)	303(60.6%)	651(65.1%)	
Unknown	92(18.4%)	82(16.4%)	174(17.4%)	
Mother’s Educational Status				
Low^a^	79(15.8%)	140(28.0%)	219(21.9%)	χ^2^ = 23.34, *p* < 0.001
High^b^	334(66.8%)	272(54.4%)	606(60.6%)	
Unknown	87(17.4%)	88(17.6%)	175(17.5%)	
Socioeconomic Status				
Low	209(41.8%)	307(61.4%)	516(51.6%)	. χ^2^ = 38.45, *p* < 0.001
High	291(58.2%)	193(38.6%)	484 (48.4%)	
School Type				
Public	212 (42.4%)	276(55.2%)	488(48.8%)	χ^2^ = 16.39, *p* < 0.001
Private	288 (57.6%)	224(44.8%)	512(51.2%)	

^a^Low- No formal education to incomplete secondary education

^b^High- completed secondary education to tertiary education. #- Likelihood ratio/Fisher exact test used because at least one cell has an expected value less than five

A significantly greater proportion of urban respondents were obese and overweight (5.4% and 11.4% respectively) compared to rural respondents (0.4% and 4.0% respectively). Similarly, more females were comparatively overweight and obese (10.2% and 3.9%) than males (5.3% and 2.0%) and more respondents who had high SES were overweight and obese (11.5% and 5.4%) compared to those with low SES (4.1% and 0.6%). The proportion of physically inactive respondents who were obese (3.8%) was almost twice those who were physically active (2.0%) and more physically inactive respondents were also overweight. A significantly higher proportion of those who have increased BP was also overweight and obese (26.8% and 19.5% respectively) compared to those who did not have increased BP (6.9% and 2.2% respectively). Similarly, there were more obese and overweight respondents among children whose parents were of high educational status compared to those whose parents had low educational status as shown in Table 2.

**Table 2:** Weight status and selected variables of total respondents

Variable	Category of Variable	Weight Status			Total (%) N = 1000	Remark
		Normal Wt (%) *n* = 894 (89.4)	Overweight (%) *n* = 77(7.7)	Obese (%) *n* = 29 (2.9)		
Location	Urban	416 (83.2)	57(11.4)	27 (5.4)	500 (100.0)	χ^2^ = 43.63 *p* < 0.001^a^
	Rural	478 (95.6)	20 (4.0)	2 (0.4)	500 (100.0)	
Sex	Male	473 (92.7)	27 (5.3)	10 (2.0)	510 (100.0)	χ^2^ = 12.29 *p* = 0.002^a^
	Female	421 (85.9)	50 (10.2)	19 (3.9)	490 (100.0)	
Age-group	Early Adolescence	403(88.5)	34(7.5)	18(4.0)	455 (100.0)	χ^2^ = 3.85^b^ *p* = 0.426
	Mid Adolescence	413(89.8)	37(8.0)	10(2.2)	460 (100.0)	
	Late Adolescence	78(91.7)	6(7.1)	1(1.2)	85 (100.0)	
Socio-Economic Status (SES)	Low SES	492 (95.3)	21(4.1)	3(0.6)	516 (100.0)	χ^2^ = 42.23 *p* < 0.001^a^
	High SES	402 (83.1)	56(11.5)	26(5.4)	484 (100.0)	
Physical Activity (MVPA +)	Physically Inactive	443(87.0)	47(9.2)	19(3.8)	509 (100.0)	χ^2^ = 6.30 *p* = 0.043^a^
	Physically Active	451(91.9.)	30(6.1)	10(2.0)	491 (100.0)	
High Blood Pressure	No	872 (90.9)	66 (6.9)	21 (2.2)	959 (100.0)	χ^2^ = 67.11^b^ *p* < 0.001^a^
	Yes	22 (53.7)	11 (26.8)	8 (19.5)	41 (100.0)	
Father’s Educational Status *n* = 826 #	Low	171(97.7)	3(1.7)	1(0.6)	175(100.0)	χ^2^ = 19.85^b^ *p* < 0.001^a^
	High	571(87.7)	59(9.1)	21(3.2)	651(100.0)	
Mother’s Educational Status *n* = 825 #	Low	210(95.9)	7(3.2)	2(0.9)	219(100.0)	χ^2^ = 16.96^b^ *p* < 0.001^a^
	High	525(86.6)	59(9.7)	22(3.7)	606(100.0)	

^a^Statistically significant

^b^Likelihood ratio/Fisher exact test used because at least one cell has an expected value < 5. #- Respondents who did not know parental educational status were excluded from analysis. **+** MVPA = Moderate to Vigorous Physical Activity (typical plus last seven days prior to interview)

The mean systolic BP gradually increased across ages 10–19 (from 93.4 to 110.5 mmHg) in rural schools and the pattern in urban schools was similar from age 10–14. The mean diastolic BP gradually increased from age 10–18 (59.0–73.2 mmHg) in rural schools but did not follow any particular trend in urban schools as there were variations across ages. The mean systolic BP for all ages combined was higher in schools located in urban than rural areas, 104.1 ± 14.71 mmHg versus 101.7 ± 12.36 mmHg. A similar result was obtained for and diastolic BP (66.8 ± 10.43 against 65.1 ± 9.30 respectively) as shown in Table 3.

**Table 3:** Descriptive Statistics of Respondents’ Blood Pressures by Age and Location

Age	Location		Mean Systolic Blood Pressure (mmHg) ± Std Deviation		Mean Diastolic Blood Pressure (mmHg) ± Std Deviation	
	Urban(%)	Rural(%)	Urban	Rural	Urban	Rural
10	39(7.8)	27(5.4)	94.5 ± 13.80	93.4 ± 8.80	60.8 ± 11.21	59.0 ± 7.87
11	53(10.6)	38(7.6)	96.4 ± 13.25	95.5 ± 11.58	61.4 ± 9.67	61.1 ± 9.05
12	54(10.8)	77(15.4)	100.6 ± 14.42	98.1 ± 11.64	54.7 ± 94.94	62.6 ± 9.04
13	84(16.8)	83(16.6)	105.0 ± 14.39	99.1 ± 10.99	66.8 ± 10.43	64.1 ± 7.86
14	98(19.6)	81(16.2)	108.4 ± 13.15	103.4 ± 12.00	70.3 ± 8.70	65.1 ± 9.10
15	72(14.4)	94(18.8)	105.0 ± 13.14	104.3 ± 12.36	68.6 ± 9.58	66.2 ± 9.43
16	63(12.6)	52(10.4)	109.6 ± 15.54	106.8 ± 12.29	70.0 ± 10.32	68.2 ± 8.97
17	24(4.8)	26(5.2)	104.5 ± 15.50	108.0 ± 11.87	64.2 ± 11.45	70.5 ± 8.73
18	11(2.2)	18(3.6)	105.4 ± 15.73	109.4 ± 11.10	67.3 ± 11.04	73.2 ± 7.58
19	2(0.4)	4(0.8)	110.0 ± 0.00	110.5 ± 13.20	75.0 ± 7.07	70.0 ± 14.14
Total	500(100.0)	500(100.0)	104.1 ± 14.71	101.7 ± 12.36	66.8 ± 10.43	65.1 ± 9.30

For the blood pressure measurements, total here refers to the mean score of all the score for the various ages

A greater proportion of respondents in rural areas (52.3% and 52.4%) had normal systolic and diastolic BP compared to those in urban areas (47.7% and 47.6%) respectively. The reverse was the case with systolic and diastolic pre-hypertension and hypertension in which respondents from urban areas had higher figures than those from rural areas. For instance, out of those who had any form of systolic hypertension, whether singly or combined with diastolic hypertension, 72% were from urban areas compared to 28.0% from rural areas. Figures for diastolic hypertension were 59.1% and 40.9% respectively. Overall, 41 respondents (4.1%) had hypertension with 65.8% of them coming from urban areas and the remaining 34.2% coming from rural areas (Table 4).

**Table 4:** Blood pressure distribution of respondents

Blood Pressure category	Urban (%)	Rural (%)	Total (%)
Normal Systolic BP	386 (47.7)	424 (52.3)	810(100.0)
Normal Diastolic BP	392 (47.6)	432 (52.4)	824 (100.0)
“Pre-Hypertension” (Systolic)	96 (58.2)	69 (41.8)	165 (100.0)
“Pre-Hypertension” (Diastolic)	95 (61.7)	59 (38.3)	154 (100.0)
“Hypertension” (Systolic)	18 (72.0)	7 (28.0)	25 (100.0)
“Hypertension” (Diastolic)	13 (59.1)	9 (40.9)	22 (100.0)
Total respondents with high BP (either systolic or diastolic or both)^a^	27 (65.8)	14 (34.2)	41 (100.0)

^a^Out of the 41 respondents who had “hypertension”, 19 had isolated systolic “hypertension”, 16 had isolated diastolic “hypertension” and six had both systolic and diastolic “hypertension”

When the BP profiles of urban and rural respondents were compared with socio-demographic and other characteristics, respondents’ BMI showed statistically significant relationships in both urban and rural areas. In urban schools, a significantly higher proportion of obese respondents (25.9%) had high BP compared to 2.9% of those with normal weight and 14.0% of those with overweight. The situation was similar in rural schools where a significantly higher proportion of obese respondents (50.0%) had high BP compared to 2.1% of those with normal weight and 15.0% of those with overweight. In both urban and rural schools, obese respondents had the highest proportion of those with high BP followed by those with overweight. A greater proportion of females (7.3%) had high BP in urban areas compared to males (3.5%), corresponding figures for rural areas were 4.5% and 1.2%. The variables “father and mother’s educational status” only showed statistically significant difference between those of low and high status in urban areas while there was no difference in rural areas. The variable sex showed a statistically significant relationship with BP in rural areas only while age-group, socio-economic status and physical activity level did not show a statistically significant difference with BP in both urban and rural areas (Table 5).

**Table 5:** High blood pressure and selected variables of urban and rural respondents

		Urban n = 500		χ,^2^ (p value)	Rural n = 500		χ,^2^ (p value)
Socio-demographic /other variables	Category	High BP			High BP		
		YES(%)	NO (%)		YES (%)	NO (%)	
Sex	Male	9(3.5)	246(96.5)	3.565 (0.059)	3(1.2)	252(98.8)	5.040 (0.025^a^)
	Female	18(7.3)	227(92.7)		11(4.5)	234(95.5)	
Age	Early adolescence	14(6.1)	216(93.9)	4.327^b^ (0.115)	8(3.6)	217(96.4)	1.755^b^ (0.416)
	Mid adolescence	13(5.6)	220(94.4)		4(1.8)	223(98.2)	
	Late adolescence	0(0.0)	37(100.0)		2(4.2)	46(95.8)	
BMI (Weight Status)	Normal weight	12(2.9)	404(97.1)	24.24^b^ (< 0.001^a^)	10(2.1)	468(97.9)	10.91^b^ (0.004^a^)
	Overweight	8(14.0)	49(86.0)		3(15.0)	17(85.0)	
	Obese	7(25.9)	20(74.1)		1(50.0)	1(50.0)	
Socio-economic Status	Low	12(5.7)	197(94.3)	0.082 (0.775)	7(2.3)	300(97.7)	0.790 (0.374)
	High	15(5.2)	276(94.8)		7(3.6)	186(96.4)	
Physical activity level	Physically inactive	15(6.2)	226(93.8)	0.618 (0.432)	10(3.7)	258(96.3)	1.841 (0.175)
	Physically active	12(4.6)	247(95.4)		4(1.7)	228(98.3)	
Father’s Educational Status n = 826 #	Low	0(0.0)	60(100.0)	7.212^b^ (0.007)^a^	5(4.3)	110(95.7)	1.241 (0.265)
	High	22(6.3)	326(93.7)		7(2.3)	296(97.7)	
Mother’s Educational Status n = 825 #	Low	1(1.3)	78(98.7)	4.257^b^ (0.039)^a^	4(2.9)	136(97.1)	0.432^b^ (0.511)
	High	21(6.3)	313(93.7)		5(1.8)	267(98.2)	

^a^Statistically significant at *p* < 0.05. Likelihood Ratio used because at least one cell has an expected value less than 5

^b^Likelihood ratio/Fisher exact test used because at least one cell has an expected value < 5. #- Respondents who did not know parental educational status were excluded from analysis

Table 6 reported findings on binary regression analysis. Among urban-based residents, significant variables associated with high BP include being overweight (OR 5.497, 95%CI 2.142–14.106, *p* = 0.0001) and obese (OR 11.783, 95%CI 4.187–33.159, p = 0.0001) while among rural residents, being females (OR 3.949, 95%CI 1.088–14.330, *p* = 0.037), overweight (OR 8.259, 95%CI 2.082–32.762, *p* = 0.003) and obese (OR 46.800, 95%CI 2.730–802.382, *p* = 0.008).

**Table 6:** Binary regression analysis of variables predicting high blood pressure among urban and rural based respondents

Variable	OR, 95%CI, p-value
Urban	
Sex (vs male)	
Female	2.167, 0.954–4.922, 0.065
Body Mass Index (vs normal)	
Obese	11.783, 4.187–33.159, 0.0001
Overweight	5.497, 2.142–14.106, 0.0001
Physically activity (vs physically inactive)	
Physically active	0.732, 0.335–1.597, 0.433
Rural	
Sex (vs male)	
Female	3.949, 1.088–14.330, 0.037
Body Mass Index (vs normal)	
Obese	46.800, 2.730–802.382, 0.008
Overweight	8.259, 2.082–32.762, 0.003
Physically activity (vs physically inactive)	
Physically active	0.453, 0.140–1.463, 0.185

On multivariate regression analysis, the significant variables associated with high BP include being female (AOR 2.067, 95%CI1.007–4.243, *p* = 0.048), overweight (AOR 5.574, 95%CI 2.501–12.421, p = 0.0001) and obese (AOR 12.437, 95%CI 4.636–33.364, p = 0.0001) (Table 7).

**Table 7:** Multivariate regression analysis of variables predicting high blood pressure among respondents

Variable	Crude OR, 95%CI, p-value	Adjusted OR, 95%CI, *p*-value
Sex (vs male)		
Female	2.611, 1.317–5.177, 0.006	2.067, 1.007–4.243, 0.048
Body Mass Index (vs normal)		
Obese	15.100, 6.031–37.801, 0.0001	12.437, 4.636–33.364, 0.0001
Overweight	6.606, 3.072–14.208, 0.0001	5.574, 2.501–12.421, 0.0001
Physically activity (vs physically inactive)		
Physically active	1.533, 0.808–2.909, 0.191	0.891, 0.451–1.759, 0.739
Residence (vs rural)		
Urban	1.982, 1.026–3.925, 0.042	1.189, 0.576–2.453, 0.639

## Discussion

This study assessed the weight status of adolescents (normal weight, overweight and obese) and related it to their blood pressure profile and confirmed that in both urban and rural areas, the weight status has a relationship with the blood pressure. Most of the overweight and obese respondents were from urban areas. The study population was chosen by multi-stage sampling across a randomly chosen senatorial district, out of three present in the State, with both rural and urban areas and private and public schools taken into consideration and thus the findings can be confidently stated as generalizable.

Both SBP and DBP generally increased with age in both urban and rural areas but especially in urban areas. This is in keeping with previous studies [21–23]. The age-specific systolic and DBP were generally higher for urban areas especially among early and mid-adolescents and the respondents in urban schools also had a significantly higher mean SBP and DBP than those in rural schools. In terms of actual prevalence of “hypertension”, respondents in urban areas constituted two-thirds of the high BP cases compared to a third from rural schools. In a Nigerian study done among adolescents the prevalence of point-hypertension was 4.6% in a semi-urban area compared to 17.5% in an urban area [10]. In another rural-urban Nigerian study, the prevalence of high BP in the urban community (9.5%) was higher than the rural community (6.3%) [24]. The rural-urban disparity has been attributed to the difference in lifestyle between urban and rural populations [10].

The combined proportion of respondents who were either overweight or obese (10.6%) showed that at least one out of every ten adolescents was either overweight or obese. The proportion of people who were overweight and/or obese is slightly higher in this study compared to similar previous studies done over the last few years in Nigeria in which the prevalence was generally less than 10 % [25, 26]. This might imply that overweight and obesity are on the increase in Nigeria as it is in most other countries of the world.

Both overweight and obesity were common in urban areas than rural areas in this study- This finding is in keeping with previous studies done in Nigeria that showed that obesity was commoner in urban schools [26, 27]. In a study done in Lagos, the prevalence rates of overweight and obesity in the urban and rural areas of Eti-osa LGA among 1504 in-school adolescents were 3.7% and 0.4%, and 3.0% and 0.0% respectively, showing a slight preponderance in urban schools [26]. In a study done earlier in rural and urban schools in Osun state, the same state as this study, using the WHO cut off points values for classifying obesity and overweight, the overall prevalence of overweight amongst 450 respondents was 3.2% with 4.1% prevalence in urban schools and 1.5% in rural schools while 0.5% of urban students were obese and no obesity was recorded in rural areas, thus overweight and obesity were commoner in urban areas [27]. Results of rural-urban studies done beyond the shores of Nigeria revealed that overweight and obesity are common in urban areas for developed/developing economies in Asia [28–30], but for western nations overweight and obesity are now commoner in rural areas [31, 32].

Both overweight and obesity were commoner in girls than boys in this study and this was statistically significant. This is in keeping with some studies that have been done previously in some developing countries [27, 28]. However, some studies have also reported a higher prevalence of overweight and obesity in boys compared to girls [16, 30]. The higher prevalence of overweight and obesity in females in this study might be due to physiological changes such as hormonal variations with respect to their age; girls tend to grow and develop secondary sexual characteristics a bit earlier than boys of the same age. This is, in fact, more likely to be true since the greater percentage of overweight and obese respondents in this study are in the early adolescent group aged 10–13 years as reported in a previous study in urban Cameroon [3]. This may be because a lot of pubertal growth spurt takes place in females during this period however further research is required.

Majority of those who were overweight and obese in this study were from high socio-economic background and parents’ having high educational status. This is in conformity with other studies that showed that overweight and obesity are common within young people in higher socio-economic group in developing countries [30, 33, 34] unlike in developed countries where it is commoner among the low socio-economic group in both urban and rural areas [35]. Similarly, more respondents who were physically active were less overweight and obese compared to physically inactive. This is in keeping with other studies which have shown that physical activity is associated with overweight and obesity, [32, 35, 36] with little or no physical activity being a predisposing factor to overweight and obesity. Overweight and obesity were found to be associated with higher systolic and diastolic BP in this study as has been found in some other studies [37–40].

From previous studies, the higher the BMI the greater the BP and this has been found in both urban and rural areas [10, 11, 41]. In a study done in Lagos, an urban area of Nigeria, higher BMI was significantly associated with hypertensive range systolic and diastolic BP [22]. Although the precise mechanisms of the positive correlation between BMI and BP are not entirely understood, factors that have been attributed include sedentariness, excessive sodium intake, hyperactivity of the renin-angiotensin and sympathetic nervous systems, insulin resistance and abnormalities in vascular structure and function [42, 43].

More females were obese and consequently had high BP than males. Unlike the finding in this study, more males had high BP in both urban and rural areas than females in a study done in Slovakia. [44] However, similar to this study, Slovakian males also had statistically higher mean values of BMI than females, thus, stressing the fact that the BP level often corresponds to the weight status. Using multi-variate analysis, the BMI (weight status) was predictive of BP in both urban and rural areas, further, confirming the association of BP with the BMI.

This study is limited in being a cross-sectional survey as no cause-effect relationship could be established. Also, some respondents did not know their parents’ educational status hence, were excluded from some analyses. However, the study findings add to the body of knowledge more information linking obesity and hypertension among in-school adolescents.

## Conclusion

BP generally increased with age with the mean systolic and diastolic BP for all ages combined higher in schools located in urban than rural areas. A little over 10 % of the respondents were either overweight or obese. A greater proportion of respondents who were overweight or obese were females, from urban areas, physically inactive, of high socioeconomic status and had high BP. The overall prevalence of high BP was 4.1%, with almost two-thirds coming from urban areas and the remaining third from rural areas. More females had high BP than males and this was significant in rural areas. BMI was significantly associated with high BP in both rural and urban areas. In both areas, especially in rural areas respondents with normal weight were less likely to have high BP compared with those who were obese.

The already documented effects of increasing weight and rising BP have been demonstrated again in this study, but this among adolescents in both rural and urban areas of a developing country. Since overweight and obesity are increasing globally, urgent measures are needed to address their increasing prevalence and the consequent high BP.

## Abbreviations

AOR: Adjusted Odd Ratio
BMI: Body Mass Index
BP: Blood pressure
CVD: Cardiovascular disease
DBP: Diastolic blood pressure
IOTF: International Obesity Task Force
LGA: Local government area
MVPA: Moderate to Vigorous Physical Activity
NCDs: Non Communicable Diseases
OAUTHC: Obafemi Awolowo University Teaching Hospitals Complex
SBP: Systolic blood pressure
SD: Standard deviation
SES: Socio-economic status
UNICEF: United Nation Children Fund
